# Early-life adversity and edentulism among Chinese older adults

**DOI:** 10.1186/s12903-022-02595-3

**Published:** 2022-11-25

**Authors:** Ziqing Tang, Chuanlong Huang, Yang Li, Ying Sun, Xin Chen

**Affiliations:** 1 Stomatologic Hospital and College, Key Laboratory of Oral Diseases Research of Anhui Province, Anhui Medical University, Hefei, China; 2 School of Public Health, Anhui Medical University, Hefei, China

**Keywords:** Deprivation, Early life adversity, Early life stress, Threat

## Abstract

**Background:**

Emerging evidence indicate the relationship between ELA with oral health problems. However, most focus on single types of adversity. The association of cumulative ELA with edentulism, the final marker of disease burden for oral health, remains unclear.

**Methods:**

Data came from 17,610 elderly participants in the China Health and Retirement Longitudinal Study (CHARLS). In 2014, the Life History Survey Questionnaire was utilized to evaluate the experience of threat and deprivation. Information on edentulism was evaluated through self-report from the follow-up in 2013, 2015, and 2018. By controlling for age, education, hukou residence, marital status, and disease history, logistic regression analyses were used to evaluate the relationships between distinct dimensions of ELA and risk of edentulism.

**Results:**

Nearly half (49.8%) of the 17,610 older persons (mean [SD] age at baseline: 63.6 [9.4] years) reported experiencing early adversity due to threat-related ELA, and 77.9% reported having deprivation-related ELA. ELA characterised by threat was associated with edentulism in both male and female participants. Two forms of threat-related ELA exposure were linked to a 1.65-fold and 1.73-fold higher risk for edentulism in both male (95% CI 1.23, 2.21) and female participants (95% CI 1.31, 2.29), compared to no threat-related ELA exposure. Both male (95% CI 2.34, 4.24) and female participants (95% CI 2.49, 4.56) had a 3.15-fold and 3.37-fold higher risk for edentulism when exposed to three or more threat-related ELAs.

**Conclusion:**

Our findings suggest that ELA marked by threat is linked to an increased risk of edentulism. The biological pathways between different dimensions of ELA and teeth loss should be clarified by future research.

## Background

Chronic psychosocial stress experienced in childhood is thought to be associated with long-term health and disease risk. In particular, early life adversity (ELA)—experiences that represent a deviation from the expected environment and require adaptation, including childhood abuse, sexual assault, neglect, and chronic poverty—create risks for lifelong chronic diseases [1, 2]. Emerging evidence indicates the relationship between ELA with oral health problems [3–6]. The Japan Gerontological Evaluation Study (JAGES) reported a significant association between the experience of abuse in childhood and the reduction of residual teeth in old age. The experience of childhood abuse increased the risk of having fewer remaining teeth by 14% [4]. A cross-sectional study among Uruguayan people aged 65–74 found that the prevalence and severity of tooth loss were positively correlated with social factors such as poor socioeconomic status, use of public health services, and frequent drinking [7]. However, most previous work focused on single types of adversity, such as physical abuse [4] or poverty [5, 6].

Poor oral health, particularly edentulism, is considered a putative risk factor for frailty [7] and mortality [8]. It is a debilitating and irreversible condition and is described as the “final marker of disease burden for oral health” [9]_._ As Sussex pointed out from a sociological perspective on the “epidemic of edentulism”, among older New Zealanders, edentulism is influenced by the combined effects of geography, economics, the dental care system and the professional culture, especially economic and social disadvantage [10]. The relationship between ELA and oral health conditions is well established. For example, periodontal disease (PD), the leading cause of tooth loss, is one of the most prevalent chronic inflammatory conditions in the world. Engel’s biopsychosocial model offers a more integrative theoretical orientation on how ELAs “get under the skin” to influence oral health outcomes. ELA induced consistent activation of the hypothalamic–pituitary–adrenal axis and the consequent release of the chronic stress hormone cortisol, placing a chronic burden on the body’s neuroendocrine and immune functions [11]. The latter contributes to an elevated oral inflammatory load, demonstrated as hyperactive neutrophils that are pivotal to periodontal tissue damage [12]. In addition, studies have shown that a number of ELA, such as poverty and childhood maltreatment, reflect a chronic situation and are linked to particular gene expression patterns, characterized by upregulated transcripts involved in inflammation and downregulated transcripts involved in antiviral responses (termed conserved transcriptional response to adversity; CTRA) [13]. This transcriptional pathway is believed to encourage chronic low-grade inflammation in response to stressful events, providing a mechanistic link between ELA and the emergence of inflammation-related illnesses [14]. In the context of oral health, immune systems dysregulated by ELA may increase susceptibilities to pathogenic bacteria and oral infection, potentially leading to tooth decay or periodontal diseases, one of the proximal causes of tooth loss.

Given that edentulism is relatively common in China with 4.5% of adults aged 65 to 74 years [15], the present study set out to identify ELA with indicators of edentulism among a representative population of 17,610 older adults from 450 villages/urban communities in 150 counties in 28 of China’s 30 provinces excluding Tibet across China, ad to provide an empirical basis for early prevention of edentulism and reduction of the health hazard effects of ELA.

We extended prior work by examining the relative associations of deprivation and threat as two forms of ELAs with edentulism. A recent conceptual model posits that two core underlying dimensions—threat and deprivation—encompass a wide variety of adverse experiences common in childhood [16–18]. Threat includes experiences involving harm or threat of harm, while deprivation involves an absence of expected inputs from the environment, such as cognitive and social stimulation. These dimensions cut across numerous adverse experiences that share the underlying experience of threat or deprivation to varying degrees. We hypothesized that ELA characterized by threat (i.e., physical abuse), in particular, would be associated with edentulism.

## Methods

### Study sample

Data was derived from the China Health and Retirement Longitudinal Study (CHARLS), which is a high quality nationally representative longitudinal survey of Chinese residents ages 45 or older and their spouses, including assessments of social, economic, and health status [19]. The baseline national wave of CHARLS was being fielded in 150 counties (or districts) and 450 villages (or resident) committees from 28 provinces (or autonomous regions, municipalities) in 2011, and three follow-up surveys were carried out in 2013 (wave 2), 2015 (wave 3) and 2018 (wave 4). Anthropometric measurements of height, weight, waist circumference, lung capacity, grip strength, speed of repeated chair stand, blood pressure, walking speed, and balance tests are conducted at every follow up wave, while blood-sample is collected once in every two follow-up cycles. All data were collected by face-to-face computer-assisted personal interviews (CAPI) with a response rate over 80% at baseline. By the time the follow-up was completed in 2018, the sample had covered a total of 19,000 respondents in 12,400 households.

Data from the baseline (2011) and follow-up waves were used in this investigation (2013, 2014, 2015 and 2018). Only male and female participants aged 50 and older at the time of the 2018 follow-up (n = 17,610; mean [SD] age at baseline, 65.3 [9.4] years) were included in our analytic sample.

### Early life adversity exposure

The Life History Survey Questionnaire, which asked individuals if they suffered any of 11 particular adversities before the age of 17, was used to analyze multiple ELA events marked by threat and deprivation in the Wave of 2014. Threat-related ELA included the following six specific adversities: (1) Unsafe community dwelling (Was it safe being out alone at night in the neighborhood where you lived as a child?); (2) Peer bullying (When you were a child, how often were you picked on or bullied by kids in your school? Is it often, sometimes, rarely or never?); (3) Female guardian physical abuse (When you were growing up, did your female guardian ever hit you?); (4) Male guardian physical abuse (When you were growing up, did your male guardian ever hit you?); (5) Sibling beat (When you were growing up, how often did your brother or sister ever hit you?); (6) Parental conflict (Did your parents often quarrel?).

Deprivation-related ELA included the following five specific adversities in childhood: (1) Biological mother absent (Before you were age 17, was it your biological mother, adopted mother or stepmother who spent the most time raising you?); (2) Biological father absent (Before you were age 17, was it your biological father, adopted father or step father who spent the most time raising you?); (3) Food scarcity (When you were a child before age 17 was there ever a time when your family did not have enough food to eat?); (4) Poor family economic conditions (When you were a child before age 17, compared to the average family in the same community/village at that time, how was your family’s financial situation?); (5) Loneliness (When you were a child, how often did you feel lonely for not having friends? Is it often, sometimes, not very often or never?).

All the ELA exposure were dichotomized into 2 categories, whether she experienced the adversity in childhood or not. Furthermore, we created threat-related and deprivation-related ELA composites by summing the total number of threat and deprivation experiences respectively and classified them as 0, 1, 2, ≥ 3 for analysis.

### Edentulism

The outcome of interest in the present study is edentulism collected in the waves of 2013, 2015 and 2018. Edentulism was measured through respondent’s report on the core question, “Have you lost all your upper and lower natural permanent teeth?” (1 = yes; 0 = no) [19].

### Covariates

Models were adjusted for age at the wave of 2018, baseline hukou residence (1 = rural, 2 = urban), baseline education level (1 = less than primary school, 2 = primary school, 3 = middle school, 4 = equal to or more than high school), marital status at the wave of 2018 (1 = married and lived with the spouse, 2 = widowed; 3 = others), disease history at the wave of 2018 (hypertension, diabetes, dyslipidemia, pulmonary disease, heart disease, kidney disease, disability, and depressive symptoms) and body mass index (BMI), of which BMI was from the physical examination questionnaire in 2015.

### Statistical analysis

Stata 16.0 was used to analyze the data. First, descriptive statistics were calculated for the demographic factors (age, gender, education, hukou residence, and marital status), edentulism, ELA categories, and disease history. Second, bivariate analysis employing Chi-square tests were used to investigate the association between edentulism prevalence and demographic factors as well as various types of ELA exposure. Third, logistic regression analyses were used to calculate the odds ratio and associated 95% confidence intervals (CIs) for various types of ELA exposure in connection to edentulism in males and females individually. We controlled for age, BMI, self-perceived health, education, hukou domicile, marital status, disease history, and threat/deprivation-related ELA in the results shown here. All statistical analyses were performed with a significance threshold of 0.05.

## Results

Descriptive statistics for key variables are presented in Table 1. A total of 17,610 older adults in China were included in the study. Nearly 15.9% of older adults with an average age of 63.6 years (SD = 9.4, ranged 50–118 years) had lost all permanent teeth. Prevalence of edentulism was higher in older age groups, and was lower in adults who are male, married and living with the spouse, as well as those living in urban areas. Participants who experienced three or more types of threat-related ELA had a higher rate of edentulism (26.0%) than participants with no deprivation-related ELA (16.1%). Older adults who experienced three or more types of deprivation-related ELA had a higher rate of edentulism (24.0%) than participants with no deprivation-related ELA (13.6%).

**Table 1:** Participant characteristics

	N	%/mean (SD)	Edentulism, %		N	%/mean (SD)	Edentulism, %
Edentulism	16,868	15.9		Deprivation	15,049	1.2 (0.9)	
Sociodemographic covariates				Biological mother absent	15,049	7.2	23.6^¶a^
Sex	17,610			1	6841	45.5	17.0^¶b^
Female	9200	52.2	17.3^¶b^	2	3430	22.8	19.3^¶b^
Male	8410	47.8	14.4	≥ 3	1455	9.7	24.0^¶b^
Education	17,610			Marital status	17,610		
< Primary	7931	45.0	22.6^¶b^	Married and lived with spouse	13,700	77.8	13.5
Primary school	3597	20.4	13.6^¶b^	Widowed	2475	14.1	31.3^¶b^
Middle school	3894	22.1	8.8^¶b^	Others	1435	8.1	11.5
≥ High school	2188	12.4	7.2	Hukou residence	17,610		
Early life adversity (W1)				Rural	10,008	56.8	19.1^¶b^
Threat	15,323	0.9 (1.1)		Urban	7602	43.2	10.1
Unsafe community dwelling	15,323	8.5	21.7^¶a^	Current health conditions			
Peer bullying	15,323	13.0	16.2	Hypertension	10,490	24.0	19.2^¶a^
Female guardian physical abuse	15,323	23.7	18.8^¶a^	Diabetes	10,490	5.9	16.7
Male guardian physical abuse	15,323	16.2	20.1^¶a^	Dyslipidemia	10,490	10.4	16.0
Sibling beat	15,323	6.2	20.5^¶a^	Pulmonary diseases	10,490	10.7	23.1^¶a^
Parental conflict	15,323	21.2	17.3^§a^	Heart diseases	10,490	12.0	20.51^¶a^
0	7628	49.8	16.1	Kidney diseases	10,490	6.7	21.9^§a^
1	3945	25.7	16.7	Disability	13,983	3.3	15.6
2	2168	14.1	18.7^§b^	Depressive symptoms	13,171	18.9	19.3^¶a^
≥ 3	1582	10.3	26.0^¶b^	Alcohol drinking	17,610	26.0	12.2
				Current smoke	17,610	27.0	15.1

^a^Compared with no adversity exposure group

^b^Compared with the lowest group

*P* < 0.05; ^§^*P* < 0.01; ^¶^*P* < 0.01

Associations between distinct dimensions of ELA and edentulism are presented in Table 2. Logistic regression analysis showed threat, instead of deprivation, was associated with edentulism in Chinese adults aged 50 and older. Two types of threat-related ELA exposure were associated with 1.65-fold and 1.73-fold higher risk for edentulism in both male (95% CI 1.23, 2.21) and female participants (95% CI 1.31, 2.29) than those with no threat-related ELA exposure; three or more threat-related ELA exposure was associated with 3.15-fold and 3.37-fold higher risk for edentulism in both male (95% *CI *2.34, 4.24) and female participants (95% *CI *2.49, 4.56).

**Table 2:** The associations between distinct dimensions of early life adversity and edentulism

Early life adversity (ELA)	Adjusted odds ratio (95%*CI*)	
	Male	Female
Threat-related ELA^a^		
0	Ref	Ref
1	1.44 (1.12, 1.86)^**§**^	1.19 (0.95, 1.49)
2	1.65 (1.23, 2.21)^¶^	1.73 (1.31, 2.29)^¶^
≥ 3	3.15 (2.34, 4.24)^¶^	3.37 (2.49, 4.56)^¶^
Deprivation-related ELA^b^		
0	Ref	Ref
1	0.85 (0.64, 1.14)	1.08 (0.83, 1.39)
2	0.82 (0.60, 1.13)	1.20 (0.90, 1.59)
≥ 3	0.97 (0.68, 1.39)	1.25 (0.90, 1.73)
Age	1.10 (1.09, 1.11)^¶^	1.09 (1.08, 1.10)^¶^

^**a**^ Adjusted for age, BMI, education, household annual income, hukou residence, marital status, disease history and deprivation-related ELA

^**b**^ Adjusted for age, BMI, education, household annual income, hukou residence, marital status, disease history and threat-related ELA

*P* < 0.05; ^**§**^ *P* < 0.01; ^**¶**^ *P* < 0.001

## Discussion

The present study investigated the association of deprivation and threatening early growth adversity ELA with edentulism in this nationally representative sample of Chinese older adults and discovered that threat, but not deprivation, was associated with edentulism. High exposure to threat-related ELA was linked to edentulism in a dose–response relationship, with a significant 3.15- and 3.37-fold increase in the risk of edentulism among older men and women, respectively.

Our finding demonstrated that 15.9% of the Chinese older population reported edentulism, which is slightly higher than national studies from US and Australia. The US Centers for Disease Control and Prevention (CDC) reported that the prevalence of edentulism in people aged 65 years and older was 12.9% in 2015–2018 [20]. Data from Australian National Adult Oral Health Study 2017–2018 reported a prevalence of 8.1% of edentulism in people aged 55–74 years [21]. However, our current finding on the prevalence of edentulism is similar with data from India (16.3%) and Russia (18%) according to the World Health Organization (WHO) Study on Global Ageing and Adult Health (SAGE) Wave 1 [22]. The higher detection rate of edentulism in the Chinese elder population compared to that of the USA and Australia might be associated with patterns of infrequent preventive dental care and lower national awareness about the importance of oral health. In addition, nearly 90% of Australians have access to fluoridated drinking water, and nearly 97% of Australian children and adults brush their teeth daily with fluoride toothpaste [23]. There is consistent evidence that community water fluoridation and the widespread use of fluoride toothpaste play the most important role in preventing dental caries in Australia [24, 25]. As dental caries is considered a major cause of tooth loss [26], caries prevention reduces the risk of tooth loss.

Our study has the following advantages, First CHARLS represents the samples of the middle-aged and the elderly, through a rigorous multi-stage probability sampling procedure to select study participants, and use effective quality control, which contains a wide range of information, meet the science related to the aging problem and the need of policy research, and in harmony with the leading international research, Ensure cross-study comparability of results [19]. Second, this survey is the first to study the relationship between ELA and edentulism through two dimensions of abuse and deprivation, which is innovative.

There are several limitations in this research. First, the self-reported nature of edentulism assessment may lead to biased information. However, previous studies have shown relatively high agreement between the self-reported and clinically-assessed number of teeth in national surveys [27]. Second, the current study obtained ELA through retrospective self-report, which is inevitably subject to recall bias. Third, detailed information on the cause and duration of tooth loss, as well as periodontal diseases or oral hygiene behaviors at different life stages, is not available in the present study. Fourth, this report only stays in quantitative research and lacks qualitative research, which needs to be further supplemented in subsequent studies. Furthermore, specific clinical data on dental health status at each life stage would be useful to investigate the mechanisms whereby ELA affects dental status In older age. Despite the limitations, the current study is the first study to elucidate the relationship between distinct dimensions of ELA and edentulism using a representative sample of adults in China.

The current study found an association between threat-related ELA, instead of deprivation-related ELA, with risk of edentulism. Most previous studies regarding the relationships between threat-related ELA and edentulism focused on single forms of ELA indicators [4]. Thus this finding is an extension of previous work considering the complex and interrelated nature of co-occurring exposure to ELA-related threats. The mechanism by which threat-related ELA causes edentulism is unclear. Recent evidence shows the association between ELA involving threats (e.g., violence exposure) with accelerated biological ageing across multiple indicators [28, 29], e.g., telomere shortening. Telomere length has been associated with impaired periodontal homeostasis and the pathophysiology of periodontitis [30], and loss of periodontal attachment has also been negatively correlated with telomere length, resulting in alveolar bone resorption [31] and possibly tooth loss.

In contrast, individuals exposed to deprivation-related ELA—including those characterized by neglect, lack of primary caregivers and chronic material deprivation (food shortage)—do not tend to exhibit a high risk for edentulism that is associated with threat. This finding is consistent with the 2012 Health and Retirement Study (HRS) in the United States demonstrating childhood financial hardship was not associated with odds of edentulism in later life [32]. Changes in social and economic position throughout the course of a person's life may have a greater impact on their oral health later in life than ELA exposure associated with deprivation. In contrast, current research suggests that people who suffer deprivation are more likely to have ongoing challenges in a number of cognitive domains, including linguistic skills and executive functioning [33, 34]. Future research on the probable mechanisms by which dental care services and teeth loss are influenced by disadvantaged childhood environments would be helpful.

The present study provides support for the association between ELA with edentulism in a representative sample of the older Chinese population. ELA prevention ought to be taken into account when developing appropriate preventive measures and treatments to enhance child welfare and parent–child connections, which may eventually aid in reducing oral health disparities. By encouraging early intervention and facilitating suitable preventive treatment, policies and initiatives seeking to reduce lifetime cumulative exposures to adversities will close the oral health gap.

## Conclusions

Our finding demonstrates that a specific dimension of ELA (i.e., threat but not deprivation) was associated with edentulism in the representative sample of older adults in China, provides preliminary evidence of biological aging in oral health effects of the different dimensions of ELA. Future research should clarify the biological pathways between different dimensions of ELA and increased risk of edentulism.

## Data Availability

Please contact China Health and Retirement Longitudinal Study (CHARLS) for data requests at http://charls.pku.edu.cn/.

## Abbreviations

ELA: Early life adversity
CHARLS: China Health and Retirement Longitudinal Study
JAGES: Japan Gerontological Evaluation Study
CDC: Centers for Disease Control and Prevention
HDI: Human Development Index
BMI: Body Mass Index
WHO: World Health Organization
SAGE: Study on Global Ageing and Adult Health
HRS: Health and Retirement Study
SD: Standard Deviation
CI: Confidence interval
OR: Odds ratio

## References

[1] Luby JL, Barch D, Whalen D, Tillman R, Belden A (2017). Association between early life adversity and risk for poor emotional and physical health in adolescence: a putative mechanistic neurodevelopmental pathway. JAMA Pediatr.

[2] Nelson CA (2017). Hazards to early development: the biological embedding of early life adversity. Neuron.

[3] Ford K, Brocklehurst P, Hughes K (2020). Understanding the association between self-reported poor oral health and exposure to adverse childhood experiences: a retrospective study. BMC Oral Health.

[4] Matsuyama Y, Fujiwara T, Aida J, Watt RG, Kondo N, Yamamoto T, Kondo K, Osaka K (2016). Experience of childhood abuse and later number of remaining teeth in older Japanese: a life-course study from Japan Gerontological Evaluation Study project. Community Dent Oral Epidemiol.

[5] Fantin R, Delpierre C, Kelly-Irving M (2018). Early socioeconomic conditions and severe tooth loss in middle-aged Costa Ricans. Community Dent Oral Epidemiol.

[6] Gomaa N, Glogauer M, Nicolau B, Tenenbaum H, Siddiqi A, Fine N, Quiñonez C (2020). Stressed-out oral immunity: a gateway from socioeconomic adversity to periodontal disease. Psychosom Med.

[7] Ramsay SE, Papachristou E, Watt RG, Tsakos G, Lennon LT, Papacosta AO, Moynihan P, Sayer AA, Whincup PH, Wannamethee SG (2018). Influence of poor oral health on physical frailty: a population-based cohort study of older British men. J Am Geriatr Soc.

[8] Kotronia E, Brown H, Papacosta AO, Lennon LT, Weyant RJ, Whincup PH, Wannamethee SG, Ramsay SE (2021). Oral health and all-cause, cardiovascular disease, and respiratory mortality in older people in the UK and USA. Sci Rep.

[9] Emami E, de Souza RF, Kabawat M, Feine JS (2013). The impact of edentulism on oral and general health. Int J Dent.

[10] Sussex PV, Thomson WM, Fitzgerald RP (2010). Understanding the ‘epidemic’ of complete tooth loss among older New Zealanders. Gerodontology.

[11] Gomaa N, Tenenbaum H, Glogauer M, Quiñonez C (2019). The biology of social adversity applied to oral health. J Dent Res.

[12] Gomaa N, Glogauer M, Nicolau B (2020). Stressed-out oral immunity: a gateway from socioeconomic adversity to periodontal disease. Psychosom Med.

[13] Cole SW (2019). The conserved transcriptional response to adversity. Curr Opin Behav Sci.

[14] Furman D, Campisi J, Verdin E (2019). Chronic inflammation in the etiology of disease across the life span. Nat Med.

[15] Jiao J, Jing W, Si Y (2021). The prevalence and severity of periodontal disease in Mainland China: data from the Fourth National Oral Health Survey (2015–2016). J Clin Periodontol.

[16] McLaughlin KA, Sheridan MA, Lambert HK (2014). Childhood adversity and neural development: deprivation and threat as distinct dimensions of early experience. Neurosci Biobehav Rev.

[17] Berman IS, McLaughlin KA, Tottenham N (2022). Measuring early life adversity: a dimensional approach. Dev Psychopathol.

[18] Ellis BJ, Sheridan MA, Belsky J (2022). Why and how does early adversity influence development? Toward an integrated model of dimensions of environmental experience. Dev Psychopathol.

[19] Zhao Y, Hu Y, Smith JP, Strauss J, Yang G (2014). Cohort profile: the China Health and Retirement Longitudinal Study (CHARLS). Int J Epidemiol.

[20] Fleming E, Afful J, Griffin SO (2020). Prevalence of tooth loss among older adults: United States, 2015–2018. NCHS Data Brief.

[21] Peres MA, Lalloo R (2020). Tooth loss, denture wearing and implants: findings from the National Study of Adult Oral Health 2017–18. Aust Dent J.

[22] Kowal P, Chatterji S, Naidoo N (2012). Data resource profile: the World Health Organization Study on global AGEing and adult health (SAGE). Int J Epidemiol.

[23] Elani HW, Harper S, Thomson WM (2017). Social inequalities in tooth loss: a multinational comparison. Community Dent Oral Epidemiol.

[24] Amarasena N, Chrisopoulos S, Jamieson LM (2021). Oral health of Australian adults: distribution and time trends of dental caries, periodontal disease and tooth loss. Int J Environ Res Public Health.

[25] Do LG, Australian Research Centre for Population Oral Health (2020). Oral Health Guidelines for use of fluorides in Australia: update 2019. Aust Dent J.

[26] Furuta M, Takeuchi K, Takeshita T (2021). 10-year trend of tooth loss and associated factors in a Japanese population-based longitudinal study. BMJ Open.

[27] Matsui D, Yamamoto T, Nishigaki M (2016). Validity of self-reported number of teeth and oral health variables. BMC Oral Health.

[28] Colich NL, Rosen ML, McLaughlin KA (2020). Biological aging in childhood and adolescence following experiences of threat and deprivation: a systematic review and meta-analysis. Psychol Bull.

[29] Drury SS, Mabile E, Brett ZH (2014). The association of telomere length with family violence and disruption. Pediatrics.

[30] Baima G, Romandini M, Citterio F (2022). Periodontitis and accelerated biological aging: a geroscience approach. J Dent Res.

[31] Steffens JP, Masi S, D'Aiuto F (2013). Telomere length and its relationship with chronic diseases—new perspectives for periodontal research. Arch Oral Biol.

[32] Purcell PJ (2012). Income replacement ratios in the Health and Retirement Study. Soc Secur Bull.

[33] Lambert HK, King KM, Monahan KC (2017). Differential associations of threat and deprivation with emotion regulation and cognitive control in adolescence. Dev Psychopathol.

[34] Miller AB, Sheridan MA, Hanson JL (2018). Dimensions of deprivation and threat, psychopathology, and potential mediators: a multi-year longitudinal analysis. J Abnorm Psychol.

